# Zinc oxide nanoparticles synthesized using *Hyssopus Officinalis* L. Extract Induced oxidative stress and changes the expression of key genes involved in inflammatory and antioxidant Systems

**DOI:** 10.1186/s40659-022-00392-4

**Published:** 2022-06-28

**Authors:** Ghasem Rahimi, Kalateh Shah Mohammad, Mahsa Zarei, Mohammad Shokoohi, Ehsan Oskoueian, Mahsa Rastegar Moghaddam Poorbagher, Ehsan Karimi

**Affiliations:** 1 Department of Biology, Mashhad Branch, Islamic Azad University, Mashhad, Iran; 2 Department of Biology, Faculty of Sciences, Hakim Sabzevari University, Sabzevar, Iran; 3 Department of Research and Development, Arka Industrial Cluster, Mashhad, Iran

**Keywords:** Green Synthesis, Toxicity, Zinc oxide, Nanoparticles, Oxidative stress

## Abstract

**Background:**

Recent advances in the synthesis of bioactive nanoparticles resulted in the discovery and introduction of new bioactive nanoparticles to the pharmaceutical industry. In this regard, this research is aimed to synthesize the zinc oxide nanoparticles (ZnO-NPs) using *Hyssopus officinalis* L. extract and to evaluate the safety of nanoparticles using Balb/C mice.

**Methods:**

Forty male mice were divided into four groups and received 0, 50, 100, and 200 mg/kg of ZnO-NPs for thirty days. At the end of the experiment, blood sugar, creatinine, aspartate aminotransferase (A.S.T.), and alanine aminotransferase (A.L.T.) were determined. Furthermore, histopathological and oxidative stress biomarker analyses in liver and kidney tissues were performed. The changes in the major inflammatory- and antioxidant-related genes were determined.

**Results:**

The results showed that blood sugar and creatinine reduced significantly (P < 0.05) when 50, 100, and 200 mg/kg ZnO-NPs were supplemented to the diet. The serum ALT and AST and lipid peroxidation in the liver and kidney tissues were increased significantly (p < 0.05) when 50, 100, and 200 mg/kg ZnO-NPs were supplemented to the diet. Supplementation of ZnO-NPs suppressed the expression of antioxidant-related genes (SOD and CAT) and up-regulated the inflammatory biomarkers (iNOS and TNF- α). The concentration of 200 mg/Kg nanoparticles indicated cellular degeneration and necrosis in the liver and kidney tissues.

**Conclusions:**

Overall, it can be concluded that supplementation of ZnO-NPs synthesized using *Hyssopus Officinalis* L. extract in this study at 50 mg/kg or higher concentrations might be toxic to the mice.

## Background

There are now numerous applications of nanotechnology in human life. It is showing promise to bridge the gap between the physical and biological sciences by applying nanostructures and nanophases to many fields of science, particularly in nanomedicine and the development of nano-based delivery systems for drugs, where the use of nanoparticles is of great [1, 2] One of the potential drug delivery vehicles are ZnO-NPs, which are semiconductor metal oxide nanoparticles and possess magnitude properties [3, 4]. They are thermally stable and have fundamental roles in proliferation, cell cycle regulation, gene expression profile, and antioxidant defense systems. Due to their small size, high surface area, high catalytic activity, and health benefits such as antibacterial, antifungal, anticorrosive, and UV filtering properties, ZnO-NPs have been extensively studied [3, 5]. As a result of their diverse application range, there is concern about their potential toxicity. The mechanism and pattern of their toxicity are therefore essential to understand [6]. Studies on the toxicity of ZnO nanoparticles have revealed oxidative stress, lipid peroxidation, cell membrane leakage, intracellular calcium increase, DNA damage, and even antiproliferative activity, which are all caused by these nanoparticles in different cell cultures. In response to a sub-lethal concentration of ZnO nanoparticles, the expression of some genes involved in apoptosis and oxidative stress is increased [7]. *In vivo* investigation of ZnO nanoparticles is vital to determine their long-term impact and possible molecular mechanisms involved in biological systems. This has been done previously, for this reason, Amara et al. illustrated the possible toxic influences of ZnO N.P.s and zinc chloride on mineral levels and biochemical parameters in rat kidney and liver through Zn (2+) ion release and this element accumulation in organs [8].

It has been indicated that plant-derived nanoparticles are low-cost, eco-friendly, and easy to acquire when compared to nanoparticles derived from physical and chemical nano synthesis.

The *Hyssopus officinalis* L. (hyssop) is one of the popular herbs in Iran and has been used traditionally for medicinal purposes. The therapeutic applications and health benefits of hyssop are mainly based on folklore rather than on scientific substantiation which renders it a valuable candidate to look for phytochemicals and probable biomedical applications [9]. Green-synthesized ZnO NPs have been used in previous studies. For example, Rahimi et al. have investigated the therapeutic effects of *Hyssopus Officinalis* extract-loaded ZnO NPs on prostate tumor cells, spermatogenesis, and testicular damage in Balb/C mice [10]. In addition, its anti-inflammatory, cytotoxicity, and anti-angiogenesis features against breast cancer cells were reported in another study [11]. However, they have not assessed its safety for potential medical applications in mice. For this reason, the current research was conducted to synthesize the zinc oxide nanoparticles (ZnO-NPs) using *Hyssopus officinalis* L. extract and to evaluate the safety of these nanoparticles using Balb/C mice.

## Result and discussion

### Serum analysis of mice treated with zinc oxide (ZnO) nanoparticles

**Fig. 1:**
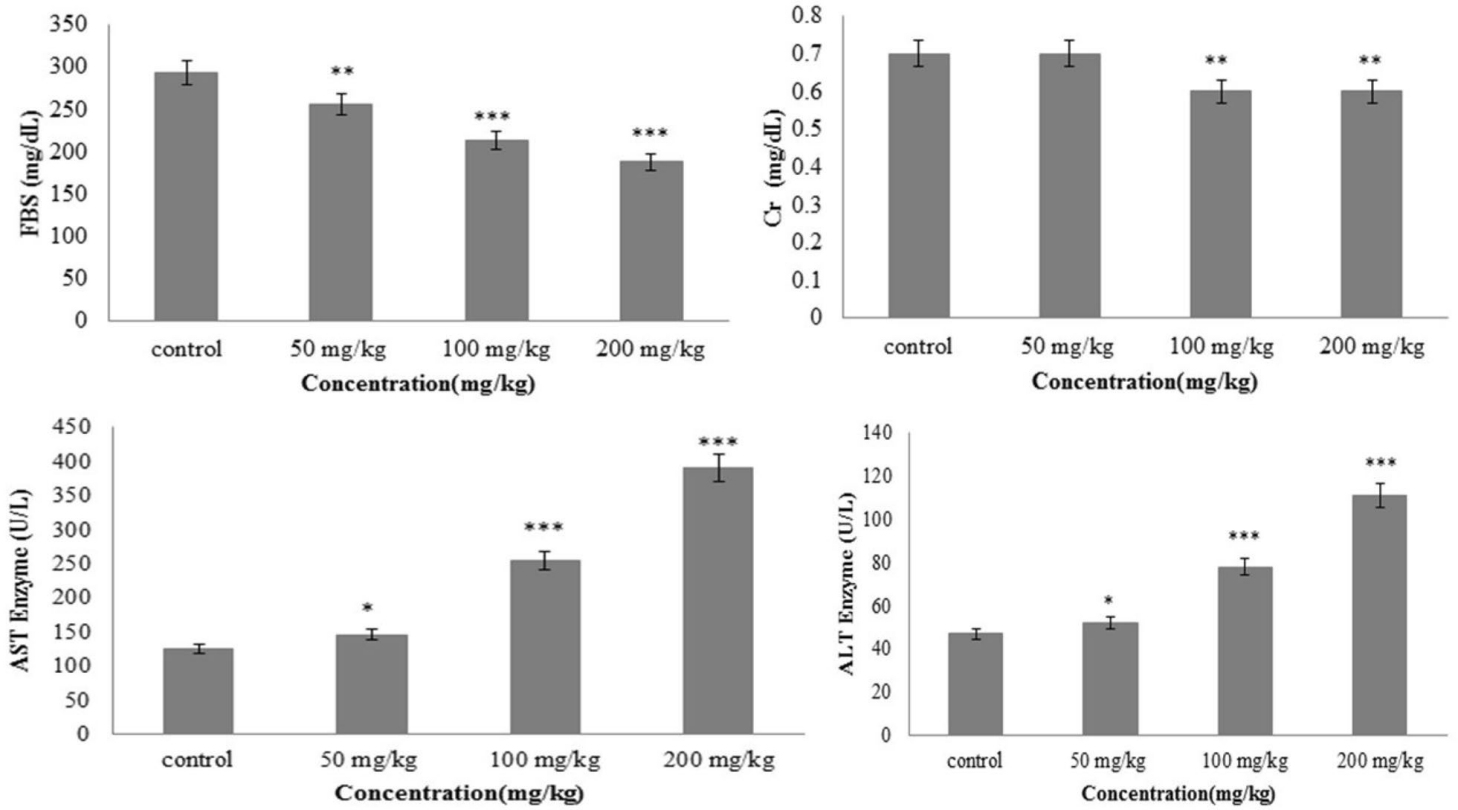
Serum concentrations of F.B.S. (Fasting blood glucose), creatinine, A.S.T. (aspartate aminotransferase), and A.L.T. (alanine aminotransferase) in experimental groups were treated with different doses of zinc oxide nanoparticles. All values indicate mean ± standard error from three separated experiments, ***P < 0.001, **P < 0.01, *P < 0.05 illustrate the remarkable difference in comparison with the control

The blood biochemical parameters are extensively utilized to monitor the response to the exogenous toxic exposure and are the fundamental biomarkers to diagnose kidney and liver malfunction [12]. The obtained results illustrated the significant differences (*P* < 0.05) in fasting blood sugar (F.B.S.) levels in all nanoparticles-treated groups 50, 100, 200 mg/kg (Fig. 1). Contrastingly, creatinine serum levels only showed a considerable difference (*P* < 0.05) in 100 and 200 mg/kg dose-treated groups in comparison with the control (Fig. 1). The overall results demonstrated that male mice treated with 100 and 200 mg/kg of ZnO-NPs showed significant decreases in the level of F.B.S. and creatinine. Figure 1 displayed a substantial positive linkage among the enhancing doses of ZnO-NPs and serum alanine aminotransferase (A.L.T.) value. Furthermore, Fig. 1 elucidated a significant positive association between serum aspartate aminotransferase (A.S.T.) levels and ZnO-NPs concentrations. Many studies have investigated the effect of herbal nanoformulations on liver and kidney function and diabetes. For instance, El-Nekeety et al. have shown that nanoencapsulation of basil essential oil did not have significant effects on A.S.T, A.L.T, creatinine, urea, and lipid profile except for HDL, which was elevated meaningfully [13]. In addition, Mohammed et al. pointed out the impact of poly(d,l)-lactic-co-glycolic acid (PLGA)-encapsulated quercetin nanoparticles on the liver and kidney’s functional biomarkers in two types of breast cancer cell lines (CAL51 and MCF7). They have exerted no significant alterations on those biomarkers so they were reported to be non-toxic [14]. Furthermore, Docosahexaenoic-acid-zinc-oxide nanoparticles were reported to decrease diabetes more effectively than free docosahexaenoic acid in diabetic rats [15]. Several studies have also illustrated the application of zinc oxide nanoparticles in nanomedicine and pharmaceutical industries [16, 17]. They demonstrated a broad range of biological potentials, including antimicrobial, antioxidant, antidiabetic, and anticancer properties. For instance, Beyrami et al. l showed the antidiabetic effect of bio-extract-mediated ZnO nanoparticles on the alloxan-induced diabetic rats. It could significantly decrease the F.B.S. levels and reduce the associated oxidative stress [18, 19]. In another study by M El-Daly h, ZnO nanoparticles decreased levels of FBS, insulin, and diminished insulin resistance in diabetic rats via increasing the expression of insulin receptor substrate-1, glucose transporters, and phosphatidylinositol 3-kinase [20]. Besides, Serum creatinine is utilized to assess renal function and it is concluded that the rate of decline in reciprocal serum creatinine might be associated with the loss of renal function [21]. For instance, Bashandy et al. illustrated a reduction in creatinine levels due to treating liver and kidney injuries induced by thioacetamide with ZnO-NPs [22]. In addition, Barakat et al. reported that treatment with ZnO nanoparticles in cisplatin-induced nephropathy rats reduced serum creatinine levels [23]. Our finding is also similar to previous experiments as there was a reduction of F.B.S. and creatinine levels in a dose-dependent manner. In addition, the enhancement in the serum levels of liver enzymes (ALT/AST) after ZnO-NPs utilization indicates cellular damage and injured hepatocytes like the results of the Ezealisiji et al. study [24]. However, in a study by El-Bahr, it is reported that the dietary ZnO nanoparticles did not affect ALT and AST concentrations in Japanese quail [25].

### Histopathological findings

**Fig. 2:**
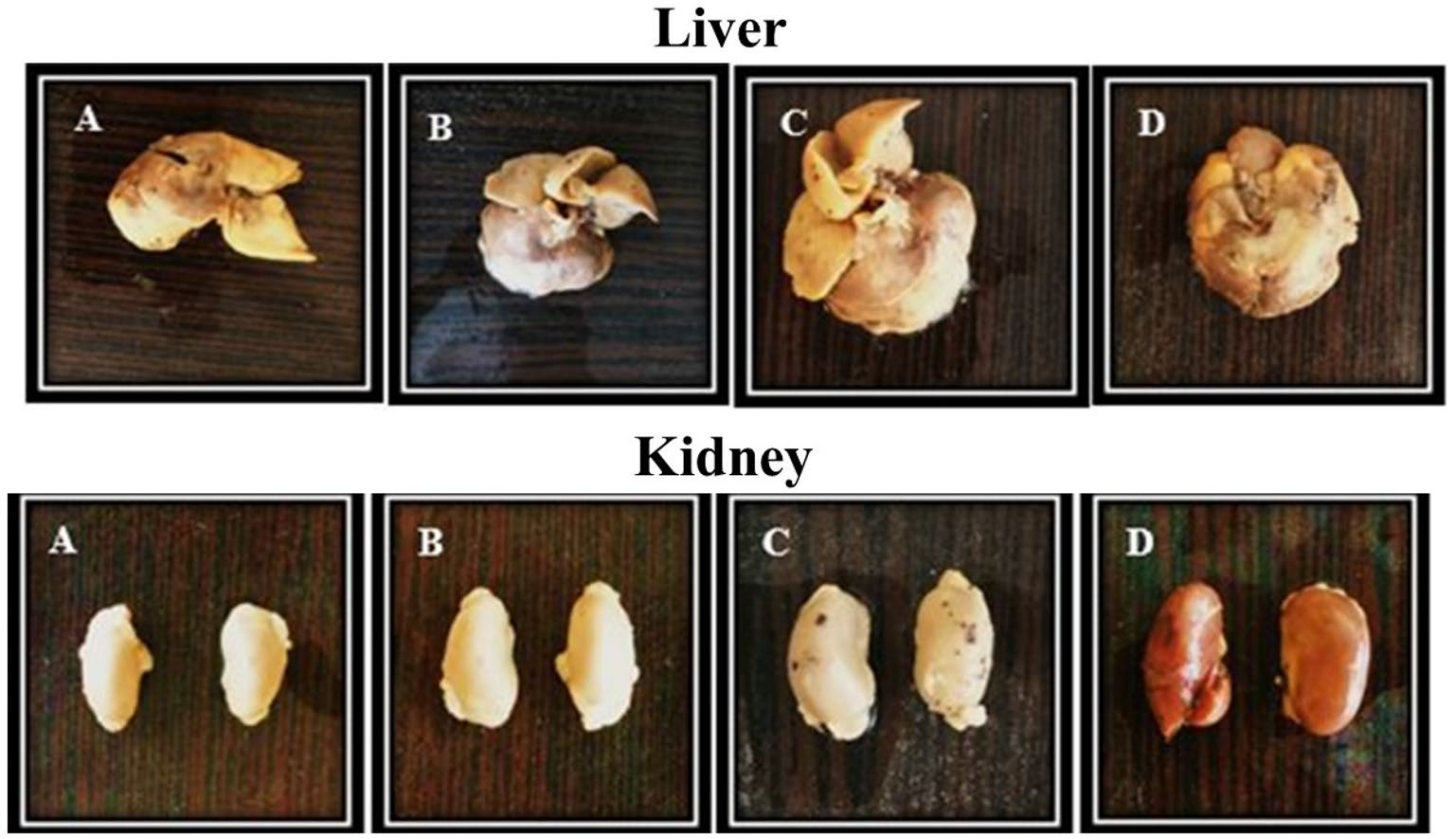
Liver and kidney tissue sample in mice; control group (**A**). In parts **B**, **C**, and **D** the mice had been treated with 50, 100, and 200 mg nanoparticles/Kg BW, respectively

**Fig. 3:**
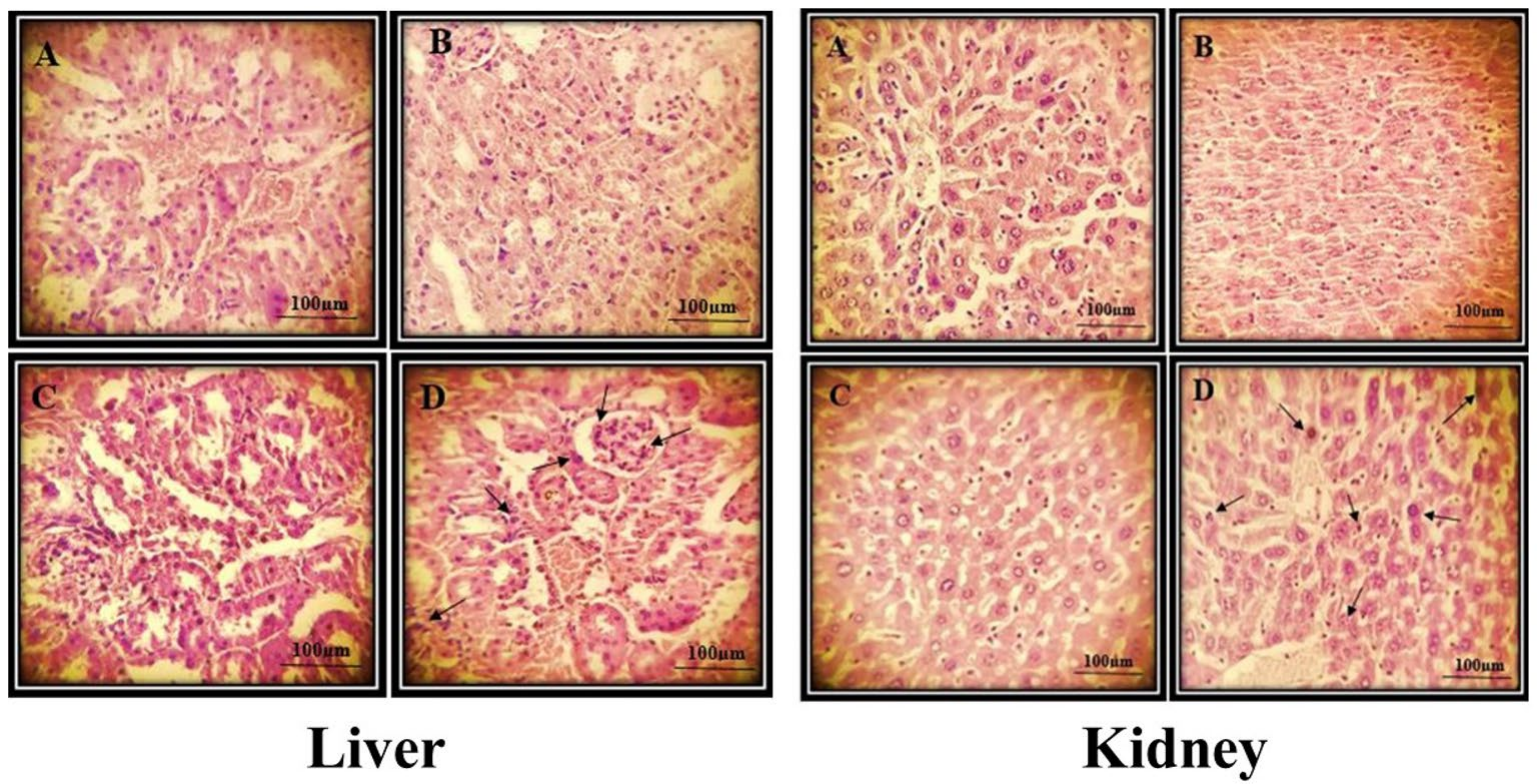
Liver and kidney histopathological properties of the mice control group (**A**). In parts **B**, **C**, and D, the mice had been treated with 50, 100, and 200 mg/Kg nanoparticles, respectively. Arrows indicated cellular degeneration and necrosis

The tissue samples and histopathological pictures of the liver and kidney between the treatment with various ZnO-NPs doses were shown in Figs. 2 and 3, respectively. The results indicated that the histopathological surveys in the liver and kidney that were treated with 50 mg/kg ZnO-NPs induced the most promising nontoxic effect as the best dose selected in this survey among other treatments with 100 mg/kg and 200 mg/kg ZnO-NPs dose, which exerted more toxic effects respectively. Arrows in the pictures indicated cellular degeneration and necrosis. Histopathological analysis of the kidney (Fig. 2) showed a canonical hepatocytes rearrangement and exhibited the cumulation of lymphocytes, hepatocytes, and intra-tubular protein deposition by raising the ZnO-NPs treatment doses. Besides that, the kidney sections from the control group and different doses of zinc oxide nanoparticles exhibited the renal cortex of the renal corpuscle with normal glomerulus. A former survey on the developed ZnO-NPs synthesized utilizing a Neem plant (*Azadirachta indica*) extract revealed that ZnO-NPs did not show any structural changes in liver and kidney and suggested descend compatibility of ZnO-NPs [26]. On the other hand, in a survey by Tang et al., which is in agreement with our results, liver and kidney cellular structures of rats that are fed with 100 mg/kg ZnO-NPs remained distinctly defined and normal, though, an increase in the dose of ZnO-NPs to 300 and 600 mg/kg led to a moderate swollen and significant swollen liver and kidney respectively. Besides, a minor hemorrhage in the liver and a severe hemorrhage in the glomerular and renal tubular epithelial cells in a 600 mg/kg ZnO-NPs dose were seen [27]. In addition, Jing Du et al., have reported that former intestinal injury could affect the ZnO nanoparticles’ toxicity in mice. They have observed that the indomethacin-induced inflammatory bowel disease (IBD) mice showed fusion of cells at the liver edge after 12 h. Among the Indo-ZnO group, some cellular degeneration and necrosis were observed in the livers and the kidneys. Mice with IBD exhibited intestinal mucosa abscission, glandular erosion, glandular atrophy, lymphocytes with inflammatory infiltration, lack of levels, fibrous tissue hyperplasia, and inflammation, all of which were more severe than those from the ZnO NP-induced mice [28]. In addition, the effects of other herbal-based nanoparticles were evaluated previously. For example, Hassanen et al. have reported the toxicopathological effect of chitosan-coated silver nanoparticles. Their Histopathological results revealed congestion, hemorrhaging, cellular degeneration, apoptosis, necrosis in liver and kidney tissue, as well as depletion of lymphocytes with an increase in macrophages in the spleen [29]. Furthermore, Sulaiman et al. have pointed out the histopatholigical effect of *hesperidin*-loaded gold nanoparticles on the liver, kidney, spleen, and lung of mice. Their results have shown no apparent damaging abnormalities in those tissues [30].

### ZnO-NPs tissue deposition

Overall, no mortality in all nanoparticle groups was observed. The average accumulation of ZnO-NPs in the liver and kidney tissues are displayed in Table 1. The serum level of zinc was increased in the liver and kidney tissues in our study as a result of ZnO-NPs treatment. Still, this elevation was only significant in 200 mg/kg of ZnO-NPs concentration with respective values of 21.5 and 20.9 ppm compared to the control. kidney, lung, and liver are probable target organs for ZnO nanoparticles’ accumulation and toxicity, which is independent of gender. Due to the newly formed Zn-S bonds, ZnO nanoparticles are absorbed in organs as ions rather than as particles. Baek et al. have reported that Zinc levels were elevated in the rats’ liver, lung, and kidney 6–24 h after receiving 50 mg/kg and 300 mg/kg ZnO nanoparticle doses, but returned to normal levels afterward. Nevertheless, when 2000 mg/kg of either ZnO nanoparticle size was administered, a high accumulation of nanoparticles in the liver and kidney within 2 and 3 days was evident but were not detected 7 days after administration [31]. Furthermore, A systematic review by Chen et al. indicated that after 24 h of ZnO-NPs treatment, the Zn content was mainly distributed in the liver and kidney [32]. In addition, Amara et al. didn’t see a significant statical increase in the Zn level of liver and kidney after treatment with 25 mg/kg of ZnO-NPs [8]. Other studies that applied herbal drug-loaded nanoparticles, showed their tissue deposition in different rat’s body organs. For instance, Said-Elbahr reported that PLGA nanoparticles with naringin and celecoxib, which have been used against lung cancer, showed high deposition potential in the liver, lung, brain, and bones [33].

**Table 1:** deposition of zinc in the kidney and liver tissues as specified by Inductively coupled plasma mass spectrometry (I.C.P.)

	Control	50 mg/kg	100 mg/kg	200 mg/kg	S.E.M
Liver (ppm)	15.3^d^	17.8^c^	19.2^b^	21.5^a^	0.34
Kidney (ppm+-)	12.5^d^	14.2^c^	17.8^b^	20.9^a^	0.18

*S.E.M* Standard error of the mean

## Gene expression analysis

### Anti-inflammatory related genes expression

Two main inflammatory biomarkers which play a significant role in the inflammatory process are TNF-α and iNOS. They have been represented to enhance the production of normal and disease-associated hepatic proteins, induce hypoglycemia [34] and adjust the expression of cell surface adhesion molecules, both on vascular endothelial cells and neutrophils [35]. The expression of iNOS and TNF-α upon various treatments with 50, 100, and 200 mg/kg of ZnO-NPs concentration are presented in Table 2. The treatment of the liver and kidney tissues with various concentrations of ZnO-NPs up-regulated the expression of these genes meaningfully (p < 0.05), ranging from 1.1 up to 3.9 folds. The up-regulation of these genes confirmed the presence of inflammation in the mice kidney and liver treated with different concentrations of zinc nanoparticles. Two other studies reached similar results as one indicated the elevation of TNF-α in the mice spleen at 12 and 24 h after treatment with ZnO-NPs. They claimed that it happened due to *in vivo* pro-inflammatory response induced by ZnO, which may be associated with oxidative stress and primary hepatic injury [36]. The other one also showed that rats treated with ZnO-NPs induced considerable elevations in the serum levels of TNF-α [37]. Furthermore, ZnO-NPs synthesized from *Allium cepa* could reinstate iNOS levels [38]. In addition, Abbasi-Oshaghi et al. illustrated that treatment with 50 and 100 mg/kg of ZnO nanoparticles in high fat diet-fed rats led to the elevation of iNOS and TNF- α gene expression [3]. Furthermore, other studies have illustrated the effect of herbal-based nanoparticles on inflammation. For instance, Azadpour et al. reported that Silymarin-PLGA nanoparticles decreased pro-inflammatory cytokines (TNF-α and IL1-β) in LPS-treated murine peritoneal macrophages [39]. In addition, Ashouri et al. have stated the reducing effect of aloe vera/chitosan nanohydrogel on the inflammatory iNOS gene expression in mice wound samples [40].

**Table 2:** The variations in the expression of various genes in the mice liver and kidney treated with a different disparate concentration of zinc nanoparticles

Items			Gene expression (Fold changes)				
			ZnO-NPs concentration (mg/kg)				
			0	50	100	200	S.E.M
Liver	Up-regulated genes						
	iNOS	1.0^c^		+ 1.4^c^	+ 1.9^b^	+ 2.7^a^	0.13
	TNF-α	1.0^c^		+ 1.6^c^	+ 2.7^b^	+ 3.9^a^	0.14
	Down-regulated genes						
	SOD	1.0^c^		− 1.5^c^	− 2.1^b^	− 2.9^a^	0.10
	CAT	1.0^c^		− 1.6^c^	− 2.3^b^	− 3.1^a^	0.12
Kidney	Up-regulated genes						
	iNOS	1.0^c^		+ 1.1^c^	+ 1.8^b^	+ 2.3^a^	0.15
	TNF-α	1.0^c^		+ 1.3^c^	+ 2.1^b^	+ 3.4^a^	0.11
	Down-regulated genes						
	SOD	1.0^c^		− 1.4^c^	− 1.7^b^	− 1.9^a^	0.10
	CAT	1.0^c^		− 1.2^c^	− 1.6^b^	− 1.8^a^	0.12

Means (n = 3) with various superscripts within a row are significantly unlike (p < 0.05).

Each gene expression was normalized to the β-actin expression as a housekeeping gene and then the result normalized to the expression of that gene in the negative control (ZnO-NPs 0 mg/kg)

*S.E.M* Standard error of the means

### Antioxidant related genes expressionAntioxidant related genes expression

Table 2 illustrates the expression analysis of S.O.D. and C.A.T. genes as significant antioxidant-associated genes in kidney and liver upon treatment with 50, 100, and 200 mg/kg of ZnO-NPs concentration. The S.O.D. and C.A.T. genes’ expression showed down-regulation (p < 0.05) upon treatment with ZnO-NPs compared to the untreated control. These antioxidant genes down-regulation confirmed the reduced cellular redox state. In line with these results, Bayat et al. have reported induction of oxidative stress in male Wistar rats livers through decreasing the gene expression of antioxidant enzymes including SOD, CAT, and GPx [41]. In contrast, in a study conducted by Barakat et al., ZnO-NPs pretreatment in the cisplatin injected rats could enhance renal antioxidant enzyme activities including S.O.D. and C.A.T., showing the ability of cell membrane integrity protection versus oxidative stress damage [23]. In previous studies, the changes in antioxidant-related genes were also assessed as a result of treatment with plant-derived nanoparticles. In this regard, Beyrami et al. revealed the upregulation of antioxidant-related genes (SOD and GPx) due to the consumption of chrysin-loaded nanoliposomes in mice’s liver challenged by cadmium [42].

### Evaluation of ZnO-NPs on the lipid peroxidation level in mice liver and kidney tissues

**Fig. 4:**
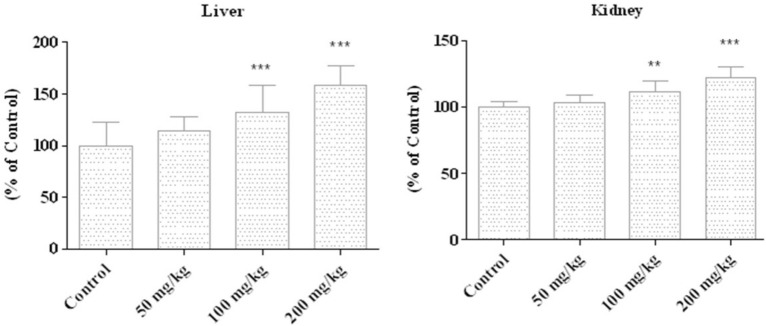
Lipid peroxidation level in mice’s liver and kidney tissues treated with 0, 50, 100, and 200 mg/kg of zinc nanoparticle. Lipid peroxidation increased significantly for two high doses. All values indicate mean ± standard error from three separated experiments, ***P < 0.001, illustrating a remarkable difference compared to the untreated control

Lipid peroxidation is an essential biomarker in free radical-mediated cell injury and plays a crucial role in human health. Free radical mechanisms in lipid oxidation are involved in the pathogenesis of human diseases like cardiovascular heart disease and cancer and the process of aging [43]. Based on the data shown in Fig. 4, the mice’s liver and kidney tissues treated with 100 and 200 mg/kg of ZnO-NPs showed a significant increase in the peroxidation index of lipids (M.D.A.) compared to the control. In this regard, Roy et al. also illustrated that ZnO-NPs increased R.O.S. generation, lipid peroxidation, and depleted antioxidant enzymes, leading to the induction macrophage cells death [44]. In contrast, EL-Bahr et al. showed that the ZnO nanoparticles’ treatment led to a reduction in the MDA levels in Japanese quail, so their findings confirm the antioxidant effects of ZnO nanoparticles [25]. This opposite result might be associated with the concentration or the synthesis technique of ZnO nanoparticles used in their study. In addition, Nouri et al. have reported that *Hyssopus officinalis L* extract has antioxidant properties, however, this feature has increased after loading its extract in nanoliposomes [45].

## Conclusions

The ZnO-NPs synthesized in this study altered the blood parameters such as fasting blood glucose, creatinine, aspartate aminotransferase, and alanine aminotransferase. The concentration of 100 and 200 mg/kg of ZnO-NPs induced inflammation and reduced the cellular redox potential in the kidney and liver tissues. The concentration of 200 mg/Kg nanoparticles indicated cellular degeneration and necrosis in the liver and kidney tissues. Overall, it can be concluded that supplementation of ZnO-NPs synthesized in this study at 50 mg/kg or higher concentration might be toxic to the mice. The toxicity of ZnO-NPs synthesized in this study might be related to the method of synthesis, therefore we recommended the evaluation of different plant materials for the synthesis of ZnO-NPs for future work.

## Materials and methods

### Chemicals and reagents

The trypan-blue, zinc oxide, tris base, triton-x, and Hepes buffer were purchased from Sigma-Aldrich Company (Saint Louis, U.S.A.). SYBR Green PCR master mix, PCR Master Mix, RNA extraction kit, and cDNA synthesis kit were from Qiagen GmbH, Hilden, Germany. The remained reagents, which are not mentioned here, were from Merck (Germany).

### **Zinc oxide nanoparticles preparation utilizing*Hyssopus officinalis*L. extract**

*Hyssopus officinalis* L. plant extract was made by locating 10 g of leaves in 100 ml Milli Q H_2_O and boiled for 2 h at 50 ^o^C. The extract was chilled at room temperature and filtered via Whatman filter paper number one. Then, the extract was kept at 4^o^C before the ZnO-NPs biosynthesis. The aqueous zinc acetate solution (50 ml, 2%) was prepared. The 20 ml of aqueous *H. officinalis* L. leaf extract (1 mg/ml) was put into an aqueous zinc acetate solution dropwise under stirring. The mixture turns yellowish, and the zinc hydroxide precipitates. The reaction was stood for 30 min for the complete reaction. The precipitate was collected using centrifugation at 15,000 g for 15 min at 4 ^o^C. Finally, the synthesized zinc nanoparticle was characterized using a particle size analyzer and scanning electron microscope (SEM) [46].

### Animal trial

All animal handling method was accomplished as per the regulations of the Islamic Azad University of Mashhad, IRAN, with their prior approval for using the animals (IR.IAU.MSHD.REC.1397.034). Moreover, working and treating animals during the study period was performed according to animal rights laws and under minimal stress. These experimental studies were performed on 40 mice (Balb/c) weighing 28–32 g used for treatment with ZnO N.P. The mice were provided from Razi Institute for Serums and Vaccines, Mashhad, IRAN, and were randomly separated into the 4 groups (0, 50, 100, 200 mg/kg/day ZnO-NPs) of ten mice each. The mice were kept in cages at 22 °C ± 1 °C and 60% ± 10% humidity with a 12-hour light period with *ad libitum* access to the commercial pelleted mice food. The mice of experimental groups received either 0, 50, 100, and 200 mg/kg of ZnO-NPs by daily oral gavage route for 30 days. After the treatments, they were anesthetized through inhaled methoxyflurane (Penthrane, Abbott Laboratories, U.K.), and the blood and the kidney and liver were collected for biochemical and histopathology studies.

### Blood biochemical parameters

The biochemical parameters in the blood were determined using 1.5 ml of blood sample collected via cardiac perforation and transferred into blood collection tubes. The centrifuge of the blood samples was done at 3000×*g* for 15 min. Furthermore, the serum was collected to determine the creatinine, F.B.S., aspartate aminotransferase (A.S.T.), and alanine aminotransferase (A.L.T.) by applying an auto-analyzer (Hitachi 902, Japan) and biochemical kits.

### Histological investigation and biopsy sampling of the mice tissue

The kidneys and livers tissues were assembled and fixed with a 10% formalin neutral buffer solution and set in paraffin. Thereupon, they cut out to 5 μm thick and used standard histopathological techniques on the glass microscope slides. Finally, the inverted microscope was applied to capture the images [47].

### Zinc tissue deposition analysis

The deposition of zinc in the kidney and liver tissues were distinguished using inductively coupled plasma mass spectrometry (ICP-MS Varian 820-MS from Varian, Australia) [48].

### Gene expression analysis

At the final stage of the experiment, the kidney and liver tissues were collected and frozen instantly in the liquid nitrogen. The R.N.A. extraction was done applying the RNeasy Mini Kit (Qiagen, Valencia, CA, U.S.A.) based on the protocol presented by the manufacturer. The reverse transcriptase PCR (RT-PCR) was accomplished for cDNA synthesis using the Qiagen cDNA synthesis kit based on the producer protocol. The real-time PCR assays were performed on a BioRad C.F.X. 96 real-time PCR thermocycler (Bio-Rad, Hercules, U.S.A.) via Quantifast SYBR green PCR Master Mix (QIAGEN, Germany). The expression of genes including tumor necrosis factor-alpha (TNF-α), inducible nitric oxide synthase (iNOS), catalase (CAT), and superoxide dismutase was analyzed. The characteristics of primers are shown in Table 3. The optimized PCR reaction status for the genes was as follows: 94 °C for 5 min (1X), then 94 °C for 25 s, then 58^o^C for 30 s, and 72^o^C for 35 s (40X). Data from the real-time PCR reactions were analyzed through C.F.X. manager software version 2 (Bio-Rad Laboratories). Each sample’s threshold cycles (Ct) were measured and normalized to β-actin (housekeeping gene). All amplifications of real-time PCR were done in triplicate.

**Table 3:** Primer specifications applied for the gene expression study

Genes		Sequences (5′ to 3′)	References
SOD	F	GAGACCTGGGCAATGTGACT	[49]
	R	GTTTACTGCGCAATCCCAAT	
CAT	F	ACATGGTCTGGGACTTCTGG	
	R	CAAGTTTTTGATGCCCTGGT	
TNF-α	F	GCCTCTTCTCATTCCTGCTTG	[50]
	R	CTGATGAGAGGGAGGCCATT	
iNOS	F	CACCTTGGAGTTCACCCAGT	[49]
	R	ACCACTCGTACTTGGGATGC	
β-actin	F	CCTGAACCCTAAGGCCAACC	[51]
	R	CAGCTGTGGTGGTGAAGCTG	

### Lipid peroxidation (M.D.A.) assay

The lipid peroxidation in the tissues was specified by malondialdehyde (M.D.A.) measurement utilizing thiobarbituric acid reactive substances. The tissues were homogenized using Ultra-Turrax homogenizer (Sigma, Germany) at 15,000 rpm for 20 s. An aliquot of 200 µl of homogenized tissue, 300 µl of distilled water, 35 µl of BHT, 165 µl of sodium dodecyl sulfate, and 2 ml of T.B.A. was transferred into the glass test tube. The mixture was heated at 90^o^C for 60 min. The solution was chilled, and 2 mL of n-butanol was added and shaken for 60 s, and centrifuged at 2000 ×*g* for 5 min. The n-butanol absorbance was recorded at 532 nm by a spectrophotometer. Results were reported as the percentage in which the malondialdehyde (M.D.A.) changes relative to the control [52].

### Statistical analysis

The data were subjected to a one-way analysis of variance, and the means were compared using Duncan’s multiple range test. The Statistical Package for Social Science (version 20; SPSS Inc, Chicago, IL) was used for this purpose. The significant Index was introduced as p ˂ 0.05 and the results were displayed as Mean ± S.D or S.E.M.

## Data Availability

The datasets applied during the current study are available on reasonable request.

## Abbreviations

ZnO-NPs: Zinc oxide nanoparticle
F.B.S: Fasting Blood Sugar
A.S.T.: Aspartate aminotransferase
A.L.T.: Alanine aminotransferase
TNF-α: Tumour necrosis factor-alpha
iNOS: Inducible nitric oxide synthase
C.A.T.: Catalase
M.D.A.: Malondialdehyde
